# From New York

**Published:** 1892-10

**Authors:** Wm. L. Russell

**Affiliations:** 151 E. 50th Street; New York


### FROM NEW YORK.

NEW YORK, September 15, 1892.

### OBSTETRICS.

During a recent lecture at the Post-Graduate Medical School Dr. E. Emory Tull, late resident physician to the Asylum for Lying-in Women, gave some simple directions for determining the position of the fœtus by abdominal palpation. More attention has been paid to this method of diagnosis during the past few years than formerly, and it is found to be of great value in many cases.

Dr. Tull's method is as follows: The patient is placed on the back, with the knees flexed, and the abdomen fully exposed. The examiner stands at one side with his face towards the patient's feet. He first lifts up the tissues composing the abdominal wall, and presses them between his fingers in order to appreciate the thickness through which he is to palpate. He then proceeds to examine the pelvic cavity to determine if it contains the head. This is done by placing the hands flatly one on each side of the uterine prominence, with the tips of the fingers far enough above the pubis to allow for the thickness of the abdominal wall already noted; firm and steady pressure is now made downward and backwards into the pelvic cavity until the fingers impinge upon the hard rounded surface of the head, or until it is made certain that no part of the fœtus is engaged in the pelvic brim. It is a cardinal rule that when any part of the fœtus can be felt filling the pelvic brim, *prior to the onset of labor*, the occiput is presenting. One pole of the fœtus having thus been located, the next point is to determine its relation to the pelvic diameters by finding the back. This is done by pressing on first one side of the abdomen and then on the other, the sides towards which the back points having a greater resistance. The diagnosis is completed by mapping out the irregular contour of the breech by grasping the fundus, the examiner now reversing his position so as to face the patient's head.

This method of examination is usually entirely satisfactory *if pursued methodically and carefully.* On this depends largely success or failure. When the abdominal wall is thick and tense some difficulty may be experienced, and the result must be corroborated by listening for the fœtal heart, and by vaginal touch. In multipara the head is not always found in the pelvis, and a malpresentation may be feared. It can usually be found just above, however, and the examination can then be continued as before.

CHOLERA.

The practical insufficiency of quarantine has been again exemplified, and cholera has slipped through and into the city. The first case appeared on the 6th inst., but after it had been investigated by the health officials, and an autopsy made with negative results, it was pronounced to be aggravated cholera morbus. Cultures made from the intestinal contents, however, revealed the spirillum of Koch, and no question of the nature of the disease remained. Five other cases have been repeated since, the source of the contagion being discovered in but two. Not one of the victims was an immigrant. It is significant, too, that the disease has almost simultaneously appeared in three sections of the city, at considerable distance from one another. These circumstances warrant the expectation that other cases will appear here and elsewhere, but it is believed that energetic isolation and disinfection will prevent a widespread epidemic.

The arrangements at quarantine have proved inadequate for the necessities of the occasion, and much unnecessary hardship has been inflicted on those detained there. The method of dealing with the ships was as follows: They were placed in the lower bay, and police boats were stationed to prevent other crafts approaching within half a mile of them. All the steerage passengers were removed to Hoffman Island, where they were bathed, and their clothing and baggage fumigated. While they were ashore the different compartments of the vessel were closed and filled with superheated steam, followed by sulphur or chlorine fumes. The passengers were then returned to the cleansed and disinfected vessel, the sick having in the meantime been removed to Swinburne Island or to a floating hospital. This method proved unsatisfactory, as cases continued to develop among the passengers or crew, rendering the vessel again an infected center. Arrangements were then made for the removal of the passengers entirely to some safe refuge, where cabin and steerage passengers would be separate from each other.

Every effort is being made by the authorities to put the city in as good hygienic condition as possible, the streets in the crowded tenement districts being kept cleaner than ever before. Inspectors of the Health Board make house to house visitations, and investigate all diarrhœa cases. A circular has been issued in which precautionary measures are suggested, which can be easily carried out. They relate principally to cleanliness, careful boiling and cooking of all substances taken into the alimentary canal, and to the avoidance of excesses or indiscretions in eating and drinking.

A Department of Pathology, Bacteriology and Disinfection has been organized by the Health Board, under the superintendence of Dr. H. M. Biggs, with Dr. Prudden as consultant. This will probably prove of great service to New York long after the cholera "scare" shall have been forgotten. Much ignorance prevails among the people concerning the nature and methods of spread of cholera and other contagious diseases, and the medical profession can do much at such a time as this to inculcate the simple rules of personal and household hygiene, the neglect of which is such an important factor in spreading them. It has been said that fright kills half the victims during a cholera epidemic, and one can readily believe this, on reading of the frenzied action of the mob at Fire Island, in preventing the landing of the cabin passengers of a quarantined vessel, on an island five or six miles from the mainland, on the ground of protecting their homes and loved ones.

### THE SPIRILLUM OF KOCH.

At a recent meeting of the Academy Section on Public Health, Dr. Sternberg, of the army, made some remarks in reference to this subject. He stated that recent investigations had shown that the micro-organism which produced cholera was not a bacillus, as was formerly supposed, but a spirillum. It had been noticed that long spirals were dependent from the comma shaped organism, in some instances, and in others that the organisms were s-shaped. These organisms were always found in the characteristic discharges of cholera patients, and had never yet been found in those of healthy persons, or in those of patients suffering from other diseases, although a large number of investigations had been made by himself and others. He thought that the prevailing opinion in regard to the slight virulence of recent dejecta was simply due to the fact that the germs had not yet been disseminated. The spirillum was found only in the contents of the intestine or in the intestinal follicles. It was seldom found in the vomited matter. It was not found in the blood or in the internal organs. The symptoms were caused by toxic albumen developed by the growth of the organisms and absorbed into the blood.

Certain facts regarding the conditions favorable to the growth or destruction of the germ are of special interest just now, when all are interested in making intelligent efforts to prevent the disease getting a foothold among us. They were stated by Dr. Sternberg as follows :

1. The spirillum grows in a variety of media, whether oxygen is present or not, that is, it is aërobic and anaërobic.

2. It will grow in any liquid alkaline in reaction, and containing a small amount of organic matter. It is destroyed by acids, although it will accommodate itself to a certain amount of vegetable acid.

3. It will survive for a considerable time, and will even grow in sterilized water and in rain water, and will remain active for sixty days in sea water.

4. A temperature of 55° C. is fatal to it.

5. It is destroyed by desiccation, and for that reason boxes and bales of new merchandise, and mail bags which may have been handled by infected persons are not very dangerous, the exposure to the air having destroyed the few germs by which they may have been contaminated. It is different, however, with rags or other articles which may have been soiled by dejecta.

6. The germs of putrefaction destroyed, the spirillum and cholera discharges lost their virulence soon after being mixed with normal fecal matter.

7. Freezing did not destroy the germ. The best method of disinfecting was by exposure to steam at 100° C., or by boiling for half an hour. The best chemicals to use were carbolic acid and chloride of lime. Two solutions should be kept at hand, the one strong and the other weak. A two per cent. solution of carbolic acid, and a one per cent. solution of chloride of lime could be used for the person, and a five per cent. solution of the one, or a four per cent. solution of the other for bedding and clothing, or discharges, etc. Bedding or clothing should be immersed for twenty-four hours.

Exposure to air destroyed the germs in three or four weeks. Exposure to air and sunlight, however, care being taken that every part of the garment was exposed, would render it safe in two or three days. Trunks, boots and other leather articles should be washed with one of the strong solutions, or destroyed. Vomited matter and dejecta should be mixed with at least an equal part of one of the stronger solutions. The dead should be immediately wrapped in a sheet soaked in one of the solutions, and then buried. He did not think it necessary to disinfect ordinary merchandise. Rags should be exposed to steam. Chloride of lime was cheaper than carbolic acid, and was effective and rapid in its operation. Caustic lime was also useful in destroying germs, although it did not destroy spores. It could be used as milk of lime, in the proportion of one part of water to eight of lime. It was important that both the lime and the chloride of lime be fresh.

THE VALUE OF SULPHUROUS ACID GAS.

At the same meeting Dr. J. H. Raymond read a paper in reference to fumigation with sulphurous acid gas, a method of disinfecting that has fallen somewhat into disrepute. He stated that the method had been pursued since at least 1000 B. C. It did not destroy spores, but it was believed that spirilla did not generate spores, and sulphur would destroy the germs themselves if properly applied. A series of experiments made by Dr. Biggs several years ago seemed to show that infected rags and other substances exposed to sulphur fumes in a vacuum for thirty minutes were rendered entirely safe.

151 *E. 50th Street.* WM. L. RUSSELL.

